# Thrombosis of the deep dorsal vein of the penis caused by vaccine-induced thrombotic thrombocytopenia: First reported case

**DOI:** 10.1080/2090598X.2022.2127236

**Published:** 2022-09-28

**Authors:** Mounir Jamali, Amine Cherraqi, Alexis Melang Mvomo, Youness Boukhlifi, Mohammed Alami, Ahmed Ameur

**Affiliations:** a Urology Department, Mohammed V Military Hospital, Rabat, Morocco; b Radiology Department, Mohammed V Military Hospital, Rabat, Morocco

**Keywords:** Thrombosis, deep dorsal vein, VITT, penis

## Abstract

The first described case of deep dorsal vein thrombosis of the penis secondary to vaccine-induced thrombotic thrombocytopenia (VITT), a complication of COVID adenoviral vector vaccines. The patient reported pain in the penis one month after vaccination. On ultrasound, a deep dorsal vein thrombosis was found and a biological workup was ordered to confirm the VITT trail. Anticoagulant therapy was immediately initiated and the patient responds well while suffering from erectile dysfunction. VITT is a potentially serious event that can be life-threatening; every practitioner should know how to deal with it.

## Introduction

The COVID-19 pandemic was one of the largest health crises the world has ever seen, killing many and affecting millions more. The advent of COVID-19 vaccines for global immunization was a major advance in the history of medicine. However, some complications have been reported, such as cardiac, neurological, cutaneous, and especially thrombotic in the context of vaccine-induced thrombotic thrombocytopenia (VITT). The mechanism of VITT is similar to that of heparin-induced thrombocytopenia (HIT), a prothrombotic disorder provoked by IgG-specific antibodies that recognize multimolecular complexes between the cationic platelet-factor 4 (PF4) and the anionic heparin and cause platelet activation through the FcγRIIA receptor [1–4].

The occurrence of venous thrombosis of the penis is often related to Mondor’s disease (superficial vein thrombosis). Several cases have been reported in the literature in recent years, while thrombosis of the deep dorsal vein of the penis has rarely been reported [5].

We report here a case suggesting vaccine-induced immunothrombotic thrombocytopenia of the deep dorsal vein of the penis.

## Case presentation

Mr. A.N., a 48-year-old health-care worker, came to our department for chronic penile pain, extending to the pelvis and perineum, exacerbated during sexual intercourse until it became unbearable and required stopping intercourse.

The pain had been evolving for two months in the context of impaired erectile function with an IIEF-5 score of 11, without any other associated signs such as discharge, hematuria, or dysuria.

The patient had no notable medical or surgical history, he did not use vasoconstrictor medications or any drugs, and did not report pelvic tumor, pelvic surgery, any trauma to the penis during intercourse, or any immobilization. However, he notified the administration of the second dose of ChAdOx1 nCoV-19 (Oxford–AstraZeneca) one month before the onset of his symptomatology.

Subsequent erectile function was normal, with an IIEF-5 score of 22.

Clinical examination found a patient in good general condition, apyretic, with spontaneous penile pain rated at 4/10 on the visual analog scale of pain and increasing to 8/10 on palpation of the penis [6].

There were no genital abnormalities such as discoloration or palpable nodularity or signs of trauma to the penis.

**Figure 1.**
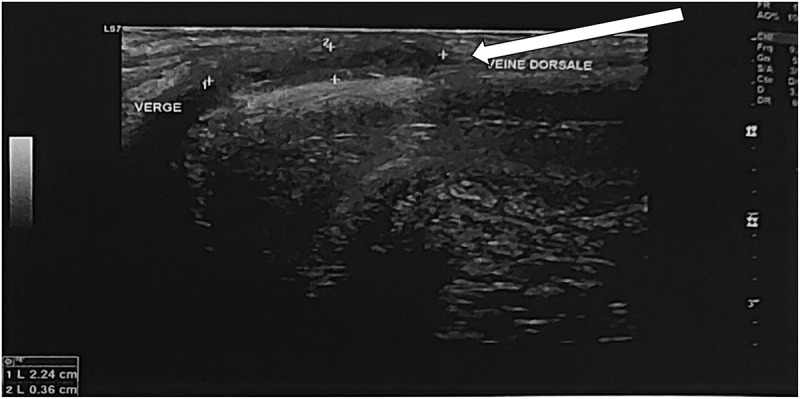
Ultrasound evaluation of the deep dorsal penile vein shows dilatation and endoluminal echogenic thrombosis measured at 3.6 mm and extended for 22 mm.

**Figure 2.**
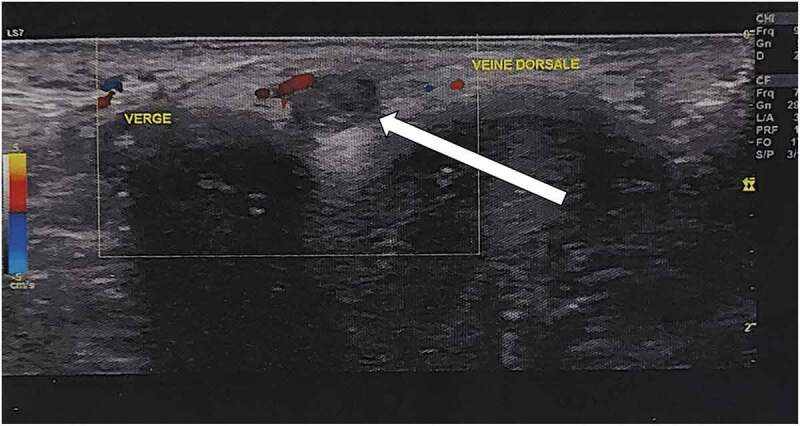
Doppler ultrasound evalution showing no flow in the vein.

A Doppler ultrasound of the penis showed a wide aspect of the deep dorsal vein of the penis with an endoluminal thrombus measured at 3.6 mm and extended for 22 mm without flow on Doppler (Figure 1, 2).

Urgent laboratory tests were ordered, with a complete blood count (CBC), D-dimer, and fibrinogen measurements (Table 1).

**Table 1.** Laboratory test results.

	Patient results
Platelets count	140.000/mm^3^
D-Dimer	1200 ug/ml (2400 FEU)
Fibrinogen	210 mg/dl
Covid PCR test	Negative
ELISA test for anti-FP4 antibodies	Not done

A PCR test for COVID-19 infection was performed, the result of which was negative.

Doppler ultrasound of the lower extremities showed no associated thrombosis of the saphenous, popliteal, superficial femoral, or external iliac veins.

The notion of recent vaccination initially suggested to us a vaccine-induced immunothrombotic thrombocytopenia, and the medical opinion of a hematologist was sought. The patient was immediately started on direct oral anticoagulation (DOAC) with a direct factor Xa inhibitor and Rivaroxaban 15 mg twice daily during the acute phase (three weeks) followed by a single daily dose of 20 mg.

The patient showed a progressive decrease in pain during the course of treatment; after one month the pain had reduced by 70% and by the third month the patient reported no pain. However, the patient had moderate erectile dysfunction.

Doppler ultrasound at three months showed no further thrombosis in the deep dorsal vein.

Long-term treatment with Rivaroxaban was well tolerated and no side effects were reported.

## Discussion

Thrombosis of the superficial venous network of the penis (Mondor’s disease) is of moderate symptomatology and may be secondary to trauma, vasculitis, hypercoagulable state, or malignancy [7,8]. Deep network involvement can lead to penile pain, priapism, and even ischemia of the corpora cavernosa. The few cases reported in the literature were secondary to COVID-19 infection, prostate abscess, or factor VIIIa hypercoagulation [5,9,10].

A few months after the launch of adenoviral vector vaccines ChAdOx1 nCoV-19 (Oxford–AstraZeneca) and Ad26.COV2. S (Janssen/Johnson & Johnson), several reports revealed the existence of vaccine-induced immunothrombotic thrombocytopenia syndrome with similarities to HIT, which results in the production of elevated levels of IgG against PF4-polyanion complexes and platelet activation responsible for thrombosis [11]. In the case of VITT, anti-PF4 antibodies could be induced by vaccine-related inflammation or by the vaccine itself, which cross-reacts with PF4 and platelets. The adenovirus used as a vector is probably involved in this pathway seeing a high affinity for PF4 and can induce platelet activation [4].

Thrombosis secondary to VITT occurs 5–28 days after administration, is often atypical, and has been reported in various veins and arteries. In the majority of cases, the site of thrombosis was the cerebral veins, while pulmonary embolism and splanchnic vein thrombosis came in second place. Other localizations, such as cardiac and peripheral venous thrombosis, were also reported [4,12,13].

The positive diagnosis is based on clinical (time to onset of symptoms), radiological (imaging showing thrombus), and biological criteria (platelet count, D-dimer assay, and ELISA test for anti-FP4 antibodies) [14]. In our case, the time to onset of symptoms was one month, the echo-Doppler test showed the thrombus, and the biological workup also supported the diagnosis, although the ELISA test was not performed because of a technical problem and the fact that the patient was late to the consultation. According to the case definition criteria published by a panel of experts in hematology, the patient fell into the category of ‘possible VITT.’

Therapeutic management has been widely recommended by learned societies and involves early and effective anticoagulation, IV immunoglobulin (IVIG), and plasma exchange or fibrinogen replacement [15]. Anticoagulation should not wait for ELISA results; the nonheparin anticoagulants fondaparinux and argatroban or a direct oral anticoagulant (such as apixaban or Rivaroxaban) is recommended if the platelet count is greater than 50,000/mm^3^ and there is no severe bleeding. IVIG is suggested in difficult cases such as bleeding or ongoing thrombus formation to inhibit platelet activation at a dose of 0.5–1 g/kg intravenously per day for two days [16]. Plasma exchange or fibrinogen replacement at > 1.0 g/L is considered only if the platelet count remains below 50,000/mm^3^ despite IVIG and corticosteroid therapy or if the fibrinogen level is below 1 g/L.

There is currently no consensus on the optimal duration of anticoagulant therapy, although some authors suggest 3–6 months. In our case, hematologists recommended three months of DOAC therapy alone due to the benign nature of the symptomatology.

VITT can be aggressive, with 20–50% of those affected dying [17]. The prognosis of VITT depends on the location of the thrombus and the severity of the syndrome (the depth of thrombocytopenia and the occurrence of hemorrhage, particularly intracranial) [14].

Our patient’s evolution was favorable under DOAC maintained for three months resulting in the disappearance of both the thrombus on the ultrasound and the pain.

The real incidence of this syndrome remains unknown, as does the natural history of the antibodies in terms of persistence and the possibility of recurrence, which would require a longer course of treatment.

## Data and material availability

Data sharing does not apply to this article as no datasets were generated or analyzed during the current study.

## Patient perspective

The patient is a health-care worker, who followed his treatment protocol rigorously.

He was slightly concerned about his erectile function and was therefore put on PDE5 inhibitors.
